# Three new species of entimine weevils in Early Miocene amber from the Dominican Republic (Coleoptera: Curculionidae)

**DOI:** 10.3897/BDJ.5.e10469

**Published:** 2017-02-03

**Authors:** Nico Franz, Guanyang Zhang

**Affiliations:** 1 Arizona State University, Tempe, United States of America

**Keywords:** Concept taxonomy, Curculionidae, Dominican amber, Entiminae, fossil, Miocene, new species, weevil

## Abstract

**Background:**

Using syntactic and semantic conventions of the taxonomic concept approach (Franz et al. 2015), we describe three newly recognized fossil broad-nosed weevils (Coleoptera: Curculionidae: Entiminae) preserved in Early Miocene amber (ca. 20.4-16.0 mya) from the Dominican Republic: *Scelianoma compacta* **sp. n.** sec. Franz & Zhang (2017) (henceforth abbreviated as [FZ2017]), *Tropirhinus palpebratus* **sp. n.** [FZ2017], and *Diaprepes anticus* **sp. n.** [FZ2017]. The taxonomic assignment of the amber inclusions is grounded in a preceding phylogenetic analysis by Franz (2012). As many as 88 of the 143 therein identified characters were coded for the fossils, whose traits are largely congruent with those present in extant congeners while also differing in ways that justify their new nomenclatural and taxonomic status.

**New information:**

We present detailed images, descriptions, and phylogenetically informed diagnoses for the three new species-level entities, along with logically consistent Region Connection Calculus (RCC-5) alignments of the amended genus-level classifications for *Scelianoma* Franz and Girón 2009 [FZ2017], *Tropirhinus* Schoenherr 1823 [FZ2017], and *Diaprepes* Schoenherr 1823 [FZ2017] - in relation to 2-4 preceding classifications published in 1982-2012. The description of *Scelianoma compacta* [FZ2017] from Hispaniola is indicative of a more widespread historical range of *Scelianoma* [FZ2017] than reflected in the extant, southwestern Puerto Rican *Scelianoma elydimorpha* Franz and Girón 2009 sec. Franz and Girón (2009). The presence of *Diaprepes anticus* [FZ2017] in Hispaniola during the Early Miocene suggests an eastward directed process of island colonization and likely speciation of members of *Diaprepes* [FZ2017], given that most extant relatives occur throughout the Lesser Antilles. The herein presented data will facilitate more reliable reconstructions of historical biographic processes thought to have played a prominent role in the diversification of the West Indian and Neotropical mainland broad-nosed weevil lineages.

## Introduction

The present study adopts the taxonomic concept approach and conventions of Jansen and Franz 2015 to consistently differentiate the following types of taxonomic name usages.

**1.** *Taxonomic concept labels* (name sec. author [year]; Berendsohn 1995) are used to identify one specific usage of a taxonomic name. Example: *Diaprepes abbreviatus* (Linnaeus 1758) sec. Franz 2010a.

**2.** Taxonomic names (without the sec. annotation) are used to refer to any or all usages associated with that name. Example: *Diaprepes abbreviatus* (Linnaeus 1758).

**3.** The term [non-focal] is added to taxonomic names whose meanings are not under scrutiny in the present context, such as names for higher-level or outgroup entities. Example: Entiminae Schoenherr, 1823 [non-focal].

For ease of legibility, we abbreviate the often appearing author specifier "sec. Franz & Zhang (2017)" with [FZ2017]. A more expansive justification for these conventions and the associated representations is provided in Franz et al. 2015, Franz et al. 2016b, Franz et al. 2016c, Franz et al. 2016a.

We newly name and describe three species-level concepts of broad-nosed weevils (Coleoptera [non-focal]: Curculionidae [non-focal]: Entiminae [non-focal] - higher-level classification in general accordance with Bouchard et al. 2011 unless stated otherwise), based on as many inclusion fossils preserved in Early Miocene (Burdigalian) amber from the Dominican Republic. Although the taxonomic novelty of these inclusions had been recognized by systematists for nearly ten years, the challenge of placing them confidently into existing classifications has precluded their formal naming and description (Steven R. Davis and Michael S. Engel, personal communication). This circumstance has changed after the publication of revisionary and phylogenetic treatments, including Franz and Girón 2009, Franz 2010a, Girón and Franz 2012, Zhang et al. 2017, and an unpublished, multi-locus molecular phylogenetic analysis of Neotropical entimine weevils with nearly 250 terminals (in preparation). In particular, novel integration of the amber-preserved specimens with the morphological cladistic analysis of reveals their genus-level identity and uniqueness in relation to the therein recognized extant lineages and terminals. Formal recognition of these fossils will promote an understanding of the historical diversity and evolutionary radiation of entimine weevils in the complex geological unfolding of the Caribbean archipelago (Ricklefs and Bermingham 2008).

## Materials and methods

### Provenance of specimen material

The herein utilized specimen material pertains to three amber inclusions on loan from two institutions that serve as their permanent repositories (Arnett et al. 1993):

**1.** Snow Entomological Museum Collection, University of Kansas, Lawrence, KS (coden: SEMC; inclusion SEMC 339)

**2.** Brodzinsky / López-Penha Collection, Department of Paleobiology, National Museum of Natural History, Washington, DC (coden: USNM; inclusions USNM505319 and USNM505325)

These inclusions pertain to the Burdigalian time period of the Early Miocene (Neogene), with an estimated age range of 20.44 ± 0.05 Ma (million years ago) to 15.97 ± 0.05 Ma (Hilgen et al. 2012). The specific mines from which the inclusions were extracted are unknown. The age and origin of Dominican amber are reviewed in Grimaldi and Engel (2005). For select references on the insect and weevil diversity reported from Dominican amber see Arillo and Ortuño (2005), Poinar (2010), Yunakov and Kirejtshuk (2011), Poinar et al. (2013), Poinar and Legalov (2015). Solórzano Kraemer et al. (2015) discuss the effect of "entrapment bias" on the taxonomic composition of amber-preserved arthropod samples.

The herein newly designated type specimens have red "holotype" labels that show the genus name and species epithet, gender symbol, author names, year, and source "sec. Franz & Zhang (2017)" (Berendsohn 1995).

### Imaging and digitization

**Imaging.** Habitus and detail photographs of the amber-included specimens were produced using either (1) a Leica M205C stereomicroscope, with an attached DFC450 camera, computer, and the Leica Application Suite (LAS) editing software, version 4.1.0.; or (2) a Visionary Digital Passport II sytem and attached Canon EOS Mark 5D II camera. An effort was made to obtain well exposed, fully focused, and scaled images; however the specific conditions of the amber inclusions - including varying optical angles in relation to the amber surface, cracks, and contaminations with non-/organic materials - made this challenging and underscores in importance of assessing the inclusions in person. The images are numbered according to their first appearance in the descriptive section.

**Digitization.** Darwin Core-compliant information and images for each of the amber-included specimens were added to the "Symbiota Collections of Arthropods Network" (SCAN) portal (see Wieczorek et al. 2012, Gries et al. 2014; URL: http://symbiota4.acis.ufl.edu/scan/). This openly accessible portal was also used to generate universally unique identifiers (UUIDs) for the specimens.

### Systematic analyses

**Morphological analysis.** Our descriptive approach follows that of preceding treatments of extant West Indian entimine weevils, in particular Franz and Girón (2009), Franz (2010a), Franz (2010b), Franz (2011), Franz (2012). Length and width measurements of the amber inclusions were taken with the Leica M205C system; however we emphasize that the measurements are approximate because the specimens' tagmata have been variously displaced or distorted in relation to a 'naturally maintained alignment', and/or deformed, by the process of fossilization. In addition, their position in the preserved amber pieces typically precludes full-length dorsal viewing access. Hence the body length was measured in lateral view, extending from the anterior margin of the eye to the posterior margin of the elytra, whereas the length of the rostrum was measured from its apex to the anterior margin of the eye. Other length measurements (rostrum, pronotum, elytra) were recorded in laternal view. Measurements of the width of the body, rostrum, pronotum, and elytra were either performed dorsally or ventrally, and often at an oblique angle.

**Phylogenetic analysis.** Inference of the phylogenetic (and hence taxonomic) identity of the specimens was greatly aided by the prior cladistic analysis of Franz (2012) (see also Franz 2014). In particular, using the Leica stereomicroscope system (magnification: 10-165x), we were able to code the amber-included specimens for as many as 88 of the 143 characters (61.5%) and corresponding states provided in Franz (2012), which in turn yields sufficiently precise inferences to place the specimens at the generic level, even in collapsed consensus topologies. The newly added codings are provided in Tables 1, 2, 3, 4, 5, 6, and the entire expanded matrix is appended in the Suppl. material 1.

In accordance with the preceding analysis (Franz 2012), the expanded character matrix was managed in ASADO (Nixon 2008) and reanalyzed using the parsimony ratchet (Goloboff et al. 2008), based on the following commands: (1) ratchet settings – 200 iterations per replication, 4% up- or down-weighted; (2) drift settings – 100 iterations per replication; (3) tree fusion settings – ten rounds, 200 MB max RAM; (4) general settings – 1000 trees to hold; (5) analyses – ratchet, drift, sectorial search, tree fusion, tree bisection reconnection (TBR)-max; and (6) xmult settings – three hits and five consense. The resulting cladograms, strict consensus tree, and character state transformations were examined in ASADO under various optimization schemes. Together with independently generated molecular phylogenetic outcomes (Zhang et al. 2017), these analyses permit recognition of both diagnostic and phylogenetic (homologous) traits of the newly recognized entities. However, the primary aim of this study is to infer the identity of the new amber-preserved specimens. A more inclusive phylogenetic reanalysis is presently underway, and thus we refrain from presenting the current phylogenetic outcomes and defer to future publications in preparation.

## Taxon treatments

### Scelianoma compacta

Franz & Zhang [FZ2017] sp. n.

http://symbiota4.acis.ufl.edu/scan/portal/collections/individual/index.php?occid=25836759

urn:lsid:zoobank.org:act:D123834D-3062-4A6E-81C1-886DD594EF11

#### Materials

- **Type status:** Holotype. **Occurrence:** catalogNumber: ARTSYS0000269; recordNumber: DR-888; recordedBy: Unknown; individualCount: 1; sex: Male; lifeStage: Adult; preparations: Amber inclusion; disposition: SEMC, on loan; otherCatalogNumbers: SEMC339; occurrenceID: 44a28738-bbf7-441d-8343-9adf009eb5b0; **Taxon:** scientificName: Scelianoma compacta; nameAccordingTo: Franz & Zhang 2017; namePublishedIn: Franz, N.M. & G. Zhang. 2017. Three new species of entimine weevils in Early Miocene amber from the Dominican Republic (Coleoptera: Curculionidae). Biodiversity Data Journal.; kingdom: Animalia; phylum: Arthropoda; class: Insecta; order: Coleoptera; family: Curculionidae; scientificNameAuthorship: Franz & Zhang, 2017; **Location:** country: Dominican Republic; stateProvince: La Vega; locality: Unknown mine; decimalLatitude: 19; decimalLongitude: -70.666667; geodeticDatum: WGS84; georeferencedBy: N.M. Franz; georeferenceSources: Google Earth; georeferenceVerificationStatus: Verfied by Curator; **Identification:** identifiedBy: N.M. Franz & G. Zhang; dateIdentified: 01/15/2017; identificationReferences: Franz, N.M. & G. Zhang. 2017. Three new species of entimine weevils in Early Miocene amber from the Dominican Republic (Coleoptera: Curculionidae). Biodiversity Data Journal.; **Event:** verbatimEventDate: Early Miocene, Burdigalian; habitat: Amber inclusion, Early Miocene (Burdigalian); **Record Level:** modified: 24/01/2017 18:31; rights: http://creativecommons.org/publicdomain/zero/1.0/; rightsHolder: University of Kansas; bibliographicCitation: Franz, N.M. & G. Zhang. 2017. Three new species of entimine weevils in Early Miocene amber from the Dominican Republic (Coleoptera: Curculionidae). Biodiversity Data Journal.; institutionID: KU SEMC; collectionID: 0acac5fe-f0ec-4d9f-82f8-0dbb74888de2; institutionCode: SCAN; collectionCode: ARTSYS; ownerInstitutionCode: KU SEMC; basisOfRecord: FossilSpecimen; source: http://symbiota4.acis.ufl.edu/scan/portal/collections/individual/index.php?occid=25836759

#### Description

**Figure 1.**
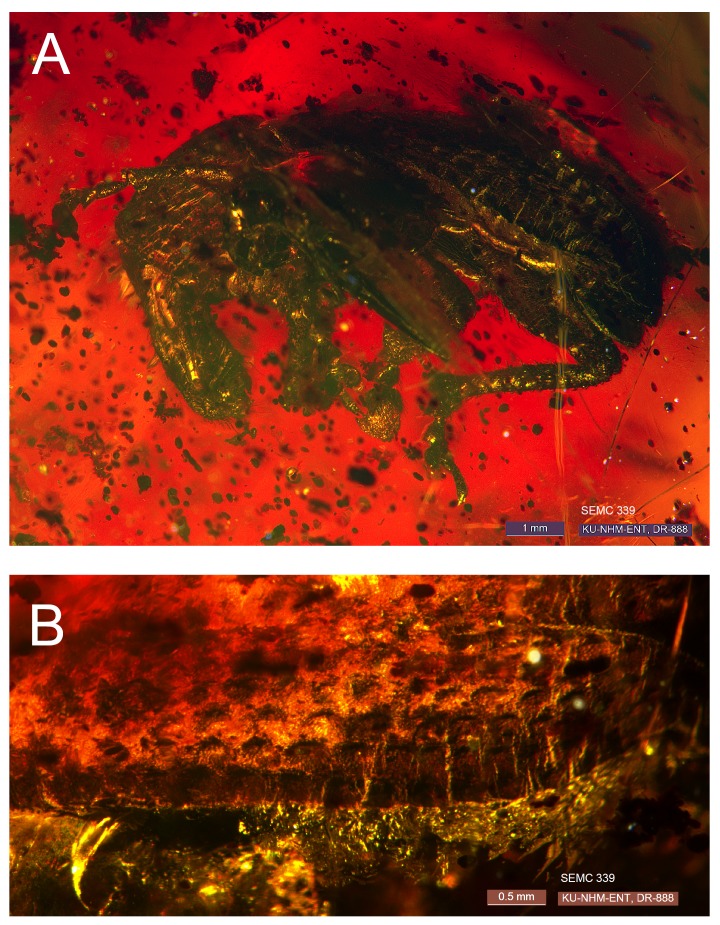
*Scelianoma compacta* Franz & Zhang 2017 **sp. n.** [FZ2017], male holotype, specimen SEMC 339 (= ARTSYS0000269). (A) Habitus, left lateral view; (B) Left elytron (incomplete). We note that optical imperfections in the amber inclusion interfere with an entire, undistorted view of the male holotype from a standard viewing angle. Similar imperfections are present in the other inclusions.

**Male - habitus (Fig. 1).** Length 9.0 mm, width 3.2 mm; shape in dorsal view elongate, length/width ratio = 2.8, widest near anterior 1/6 of elytra; shape in lateral view slightly compressed. Integument uniformly dark (black); surface sculpture of pronotum and elytra homogeneously foveate to lacunose, with deep, densely and regularly arranged, subcircular impressions, otherwise rugulose; integument covered with setae and scales, each most apparent on rostrum and legs; setae regularly arranged, dark brown, short and recurvate; scales circular and apparently densely arrangement and light to dark brown, though not well preserved in the type specimen.

**Mouthparts.** Mandibles equilateral, with 5-8 fine setae; mandibular scar positioned apicolaterally, projected. Maxillae with maxillary palps apparently 3-segmented (2 projecting palpomeres visible). Labium with prementum cordate, slightly wider than long; labial palps apparently 3-segmented.

**Rostrum.** Length 1.85 mm, rostral/pronotal length ratio 0.6, rostral length/width ratio 2.0 (though rostrum laterally compressed due to fossilization). Rostrum in dorsal view rectangular, anteriorly widened, anterodorsal margin weakly emarginate; epistoma with nasal plate (see Vaurie 1963) slightly angled in relation to posterior rostrum region, depressed, V-shaped; dorsal surface with a deep, median sulcus. Rostrum in lateral view anteriorly slightly expanded, occipital sutures extending to subapex and visible; scrobe lateral, nearly extending along entire rostrum though terminating anteriad of eye; antennal insertion near anterior 1/3. Antennae 11-segmented; scape extending to posterior margin of eye, and passing over eye (in idealized position); funicle 7-segmented, funicular antennomeres elongate, clavate; club 3-segmented, small, similar in length to funicular antennomeres V-VII.

**Head.** Eyes small, globular (strongly roundly protruded), laterally positioned, separated by distance shorter than anterior-to-posterior length of each eye; outline in lateral profile elliptical (horizontally more extended), anterior and posterior margins more strongly rounded, and with an anteocular invagination (see Franz 2012) extending from anteroventral to dorsoventral edge of eye. Head not constricted posteriad of eyes.

**Thorax.** Pronotum in dorsal view elongate-tubular, length/width ratio 1.5, pronotal/elytral length ratio 0.43; widest near mid point; surface foveate to lacunose (including lateral regions), lacunae somewhat variable in size and arranged in an off-set, honeycomb-like pattern; median sulcus absent. Pronotum in lateral view tubular, slightly arcuate; anterolateral margins straight (without postocular lobe), postocular vibrissae absent. Scutellum exposed by elytra, small, subcircular. Epipleura challenging to observe (distorted, obscured), though apparently similar to those of *Scelianoma elydimorpha* sec. Franz and Girón (2009). Prothoracic ventrite with anterior transverse sulcus; procoxal cavities positioned near mid point, contiguous. Mesothoracic ventrite with plumose-scopiform scales; mesocoxal cavities narrowly separated. Metathoracic ventrite challenging to observe; metacoxal cavities widely separated. Metendosternite not observed.

**Legs.** Prothoracic and metathoracic legs each longer than mesothoracic legs, generally similar to those of males of *Scelianoma elydimorpha* sec. Franz and Girón (2009). Profemoral/pronotal length ratio 0.8; profemur ventrally inermous. Protibial/profemoral length ratio 1.1; protibia slightly arcuate, apically exanded; anteromesal margin with 5-8 roundly triangular teeth, size increasing apically, each tooth distally with 1 brownish, spiniform seta; protibial apex with anterior margin truncate, setal comb absent, promucro similar in length to protarsal claw; protarsus with tarsomere I longer than II; II and III similar in length and jointly as long as V; protarsal claws paired, separate, simple. Mesotibiae and metatibiae nearly straight, apically obliquely truncate; metatibial apex with a narrow (linear) outer bevel ("corbel enclosed"; see Thompson 1992).

**Elytra (Fig. 1).** Length/width ratio 2.2; widest near anterior 1.4; anterior margins jointly minimally wider than posterior margin of pronotum, nearly straight; humeri absent; lateral margins slightly angulate: diverging along anterior 1/6, subrectate and slightly converging along posterior 1/6; posterior margins narrowly rounded. Elytra in lateral view with dorsal outline subplane along anterior 5/6, posterior 1/6 with distinctly angled, straight declivity, mesal elytral margin projected along angulation. Elytra with striae I-IX complete, stria X incomplete (challenging to observe); striae wider than intervals; punctures large, deep, foveate to lacunose, subcircular to elliptical, and arranged from stria to stria in an off-set, honeycomb-like pattern; intervals slightly elevanted and rounded.

**Wings.** Absent.

**Abdomen.** Venter with only abdominal ventrites VI and VII visible (and displaced by process of fossilization), each similar in length, and VII with posterior margin widely rounded. Pygidium entirely covered by elytra.

**Terminalia.** Terminalia not unambiguously observed; however, located just to the left side of the amber-included specimen are several displaced, distorted chitinous structures that apparently include the male spiculum gastrale and median lobe in more or less parallel orientation to the remainder of the specimen. Accordingly (with aforementioned caveats), the presumed spiculum gastrale is similar to that of *Scelianoma elydimorpha* sec. Franz and Girón (2009), and slightly shorter than the median lobe which narrowly linear in dorsal view and narrow and straight in lateral view, though more arcuate (inflected) along posterior 2/5, with dorso- and ventrolateral margins posteriorly continuously converging, and terminating in a narrowly rounded apex.

**Female.** Unknown.

#### Diagnosis

**Generic placement.** *Scelianoma compacta* [FZ2017] shares with *Scelianoma elydimorpha* Franz and Girón sec. Franz and Girón (2009) numerous phylogenetically informative traits inferred in Franz (2012) that substantiate this generic placement (see also Franz and Girón 2009 and Tables 1, 2, 3, 4, 5, 6). They include: character 18(1): rostrum with one dorsal, median sulcus; character 23(0): scrobe (of antenna) passing over eye in lateral profile; 26(1) rostrum (ventral side) with occipital sutures anteriorly extending to subapex of rostrum, anteriorly ascending and visible in lateral profile; 34(1): head with an anteocular invagination, extending from anteroventral to anterodorsal edge of eye; 35(1): head with eyes in dorsal profile entirely positioned on lateral surface of head; 58(2): metatibial apex with an outer bevel ("corbel enclosed"; see Thompson 1992); 62(1): elytra with humeri absent; 66(1): elytra with declivity in lateral profile strongly angulate (Franz and Girón 2009); and 83(1): wings not developed (absent). This combination of character states is shared only between *Scelianoma compacta* [FZ2017] and *Scelianoma elydimorpha* sec. Franz and Girón (2009), rendering them monophyletic and thus congeneric in our expanded analysis.

Close extant relatives of *Scelianoma* Franz and Girón [FZ2017] include members of *Artipus* Sahlberg sec. O'Brien and Wibmer (1982) that frequently display a wider shape, a wider and dorsally more narrowly (and non-continuously) sulcate rostrum (character 40[1] of Franz 2012), with the scrobe and hence the antennal scape passing ventrad of the eye in lateral profile (and idealized position), and characteristically irregular (punctate to linear) dorsal pronotal foveae (character 43[1] of Franz 2012). We note, however, that *Artipus* sec. O'Brien and Wibmer (1982) is likely not a monophyletic, comprehensively sampled entity, and indeed a revision of this entity is underway (N.M. Franz, in preparation). The revision is unlikely to alter present inferences regarding the identity of *Scelianoma* [FZ2017].

*Scelianoma* [FZ2017] is also distinct from other extant Caribbean groups such as *Apotomoderes* Dejean sec. Franz (2010b) and *Melathra* Franz sec. Franz (2011) which have a head with a conspicuous postocular constriction (character 32[1]), a profemoral tooth (character 52[1]), and a less abruptly angulate elytral declivity (character 66[0]); all in accordance with Franz 2012).

Franz and Girón (2009) preferred a placement of *Scelianoma* sec. Franz and Girón (2009) in the tribe Eustylini Larcordaire 1863 [non-focal], but this was not supported in Franz (2012). We defer to future studies to assess the validity of this tribal placement.

**Differential diagnosis.** *Scelianoma compacta* [FZ2017], in addition to being extinct and recorded from Dominican amber, is differentiated from the extant, southwestern Puerto Rico-inhabiting *Scelianoma elydimorpha* sec. Franz and Girón (2009) by having a smaller size and less elongate body form, a more strongly foveate to lacunose dorsal sculpture on the pronotum and elytra, and a more slender rostrum, although the latter appears to have been laterally compressed by the process of fossilization. If our interpretation of the Terminalia of *Scelianoma compacta* [FZ2017] is valid, then then the posterior region of the median lobe is less arcuate in this species than in *Scelianoma elydimorpha* sec. Franz and Girón (2009).

#### Etymology

The epithet - "thick, firm, compact" (Brown 1956) - refers both to the shorter, more compact habitus of *Scelianoma compacta* [FZ2017] in comparison to *Scelianoma elydimorpha* sec. Franz and Girón (2009), and to the 'compressing and distorting' effect that the fossilization process appears to have had on the specimen.

#### Distribution

*Scelianoma compacta* [FZ2017] is known only from the examined Dominican amber inclusion ("SEMC 339"; see Material) of the Burdigalian time period. The specific mine of origin for this inclusion is unknown.

#### Ecology

Unknown.

### Tropirhinus palpebratus

Franz & Zhang [FZ2017] sp. n.

http://symbiota4.acis.ufl.edu/scan/portal/collections/individual/index.php?occid=25836760

urn:lsid:zoobank.org:act:2E8D32B1-D021-4E02-BABE-B494098D4C94

#### Materials

- **Type status:** Holotype. **Occurrence:** catalogNumber: ARTSYS0000270; recordNumber: Woodruff #9768; recordedBy: R.E. Woodruff; individualCount: 1; sex: Female; lifeStage: Adult; preparations: Amber inclusion; disposition: USNM, on loan; otherCatalogNumbers: USNM505319; occurrenceID: 266d8782-5bf5-4763-b3fa-ea057a3fc55a; **Taxon:** scientificName: Tropirhinus palpebratus; nameAccordingTo: Franz & Zhang 2017; namePublishedIn: Franz, N.M. & G. Zhang. 2017. Three new species of entimine weevils in Early Miocene amber from the Dominican Republic (Coleoptera: Curculionidae). Biodiversity Data Journal.; kingdom: Animalia; phylum: Arthropoda; class: Insecta; order: Coleoptera; family: Curculionidae; scientificNameAuthorship: Franz & Zhang, 2017; **Location:** country: Dominican Republic; stateProvince: La Vega; locality: Unknown mine; decimalLatitude: 19; decimalLongitude: -70.666667; geodeticDatum: WGS84; georeferencedBy: N.M. Franz; georeferenceSources: Google Earth; georeferenceVerificationStatus: Verfied by Curator; **Identification:** identifiedBy: N.M. Franz & G. Zhang; dateIdentified: 01/15/2017; identificationReferences: Franz, N.M. & G. Zhang. 2017. Three new species of entimine weevils in Early Miocene amber from the Dominican Republic (Coleoptera: Curculionidae). Biodiversity Data Journal.; **Event:** verbatimEventDate: Early Miocene, Burdigalian; habitat: Amber inclusion, Early Miocene (Burdigalian); **Record Level:** modified: 24/01/2017 18:31; rights: http://creativecommons.org/publicdomain/zero/1.0/; rightsHolder: United States National Museum; bibliographicCitation: Franz, N.M. & G. Zhang. 2017. Three new species of entimine weevils in Early Miocene amber from the Dominican Republic (Coleoptera: Curculionidae). Biodiversity Data Journal.; institutionID: USNM; collectionID: 0acac5fe-f0ec-4d9f-82f8-0dbb74888de2; institutionCode: SCAN; collectionCode: ARTSYS; ownerInstitutionCode: USNM; basisOfRecord: FossilSpecimen; source: http://symbiota4.acis.ufl.edu/scan/portal/collections/individual/index.php?occid=25836760

#### Description

**Figure 2.**
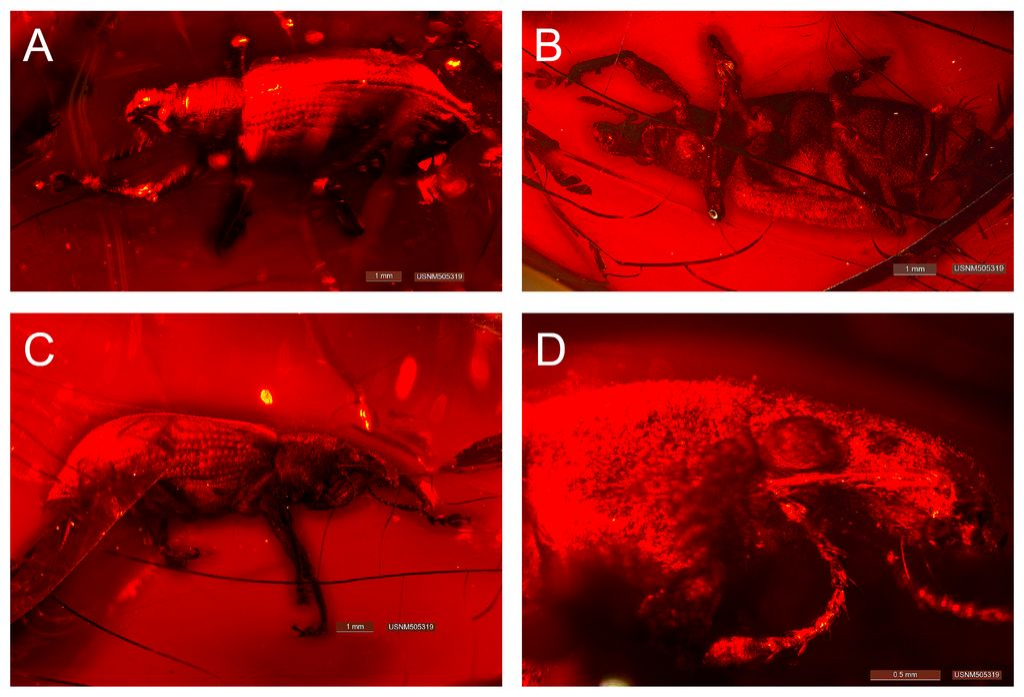
*Tropirhinus palpebratus* Franz & Zhang 2017 **sp. n.** [FZ2017], female holotype, specimen USNM505319 (= ARTSYS0000270). (A) Habitus, dorsal view; (B) ventral view; (C) right lateral view; (D) head and pronotum, lateral view.

**Female - habitus (Fig. 2).** Length 8.6 mm, width 3.6 mm; shape in dorsal view oval to elongate, length/width ratio 2.4, widest near mid region of elytra; shape in lateral view elongate to pyriform. Integument uniformly dark brown to black; surface sculpture punctate, subcircular punctures largest on pronotum; integument entirely and homogenously covered with setae and scales, each apparently pale yellow in color, with no maculae apparent, darker and with metallic aspects on the legs; setae short and linear, densely and regularly arranged, directed posteriad, appressed, scales very small, subcircular, overlapping.

**Mouthparts.** Mandibles equilateral, with 3-5 coarse and several finer setae; mandibular scar positioned apicolaterally, projected. Maxillae not apparent, covered by labium (however, the maxillary palps are 3-segmented in extant members of *Tropirhinus* Schoenherr [FZ2017]). Labium with prementum cordate, equilateral; labial palps apparently 3-segmented.

**Rostrum.** Length 1.3 mm, rostral/pronotal length ratio 0.8, rostral length/width ratio 1.1. Rostrum in dorsal view equilateral to rectangular, dorsolateral margins nearly straight and distance between them anteriorly gradually widening, anterodorsal margin with a distinct, narrow, V-shaped mesal emargination; epistoma with nasal plate (see Vaurie 1963) weakly developed, angled in relation to posterior rostrum region, slightly depressed, V-shaped carina absent; dorsal surface with a median, wide, glabrate, weakly projected carina (or elevation), extending from posterior margin of epistoma to mid point between eyes. Rostrum in lateral view slightly arcuate, tumescent in dorsal mid region; scrobe lateral, slightly arcuate, posteriorly directed ventrad of eye, though also terminating anteriad of eye; antennal insertion near anterior 1/4. Antennae 11-segmented; scape slender, slightly arcuate, extending to posterior margin of eye, and passing ventrad of eye (in idealized position); funicle 7-segmented, funicular antennomeres elongate, weakly clavate, I and II similar in length, III to VII shorter, and increasingly so towards the apex; club 3-segmented, narrowly elongate, similar in length to funicular antennomeres V-VII.

**Head.** Eyes large, globular (strongly roundly protruded), dorsolaterally positioned, separated by distance similar to anterior-to-posterior length of each eye; outline in lateral profile elliptical (horizontally more extended), ventral margin less rounded.

**Thorax.** Pronotum in dorsal view equilateral to transverse, weakly convex, length/width ratio 0.85, pronotal/elytral length ratio 0.25; widest near posterior 1/3, lateral margins continuously rounded; surface punctate, with a wide, elliptical median sulcus (or impression) extending along anterior 1/2 of pronotum. Pronotum in lateral view equilateral; anterolateral margins with a small postocular lobe, and dorsad thereof with a tuft of 4-6 slightly longer, anteriorly directed setae ("postocular vibrissae", except these are not projecting from the postocular lobe but are dorsad of it). Scutellum exposed by elytra, small, escudate, posterior margins rounded. Epipleura with mespisternum triangular; mesepimeron dorsally oblique truncate; metepisternum linear, anteriorly widened; metepimeron entirely covered by elytron. Prothoracic ventrite with anterior margin widely emarginate; proxocal cavities positioned near mid point, contiguous. Mesothoracic ventrite with plumose-scopiform scales; mesocoxal cavities separeated by distance 1/3 as wide as each mesocoxal cavity. Metathoracic ventrite with median sulcus present as a large, transverse fovea positioned anteriad of posterior margin; metacoxal cavities separated by distance similar to width of each metacoxal cavity. Metendosternite not observed.

**Legs.** Prothoracic and metathoracic legs each slightly longer than mesothoracic legs (mesofemora shortest in comparison), generally similar to those of *Diaprepes abbreviatus* sec. Franz (2010a). Profemoral/pronotal length ratio 1.3; profemur ventrally inermous. Protibial/profemoral length ratio 1.2; protibia apically angulate-arcuate, width similar throughout; anteromesal margin with 5-8 small, triangular teeth, each tooth distally with 1 brownish, spiniform seta; protibial apex with anterior margin truncate, setal comb absent, promucro similar in length to protarsal claw; protarsus swith tarsomeres I and II similar in length, each slightly shorter than III which in turn is shorter than V; protarsal claws paired, separate, simple. Mesotibiae and metatibiae nearly straight, apically slightly expanded and obliquely rounded; metatibial apex with a narrowly elliptical outer bevel ("corbel enclosed"; see Thompson 1992).

**Elytra.** Length/width ratio 1.8; widest near mid region; anterior margins jointly wider than posterior margin of pronotum, slightly sinuate; humeri present, rounded; lateral margins continously rounded, nearly straight in mid region, more strongly converging in along posterior 1/4; posterior edges each with a short, narrowly triangular, ante-apical projection. Elytra in lateral view with dorsal outline subplane along anterior 1/2, thereafter continuously rounded (hence declivity convex), less so along posterior 1/8. Elytra with striae I-IX complete, stria X only apparent along anterior and posterior 1/3; striae similar in width to intervals; punctures separated by distance similar to width of each puncture; intervals slightly and roundly elevated; pale-colored scales and setae covering elytra homogenously, with no maculae apparent.

**Wings.** Present, yet not observed (covered by elytra).

**Abdomen.** Venter with segments III and IV jointed (see Thompson 1992 for segment homology), similar in length, and separated by sinuate suture; V-VII separate; V and VI jointly slightly shorter than IV, posterior margins elevated-projected; VII similar in length to IV, triangular, posteriorly narrowly truncate. Pygidium posteriorly narrowly rounded, covered by elytra.

**Terminalia.** Not unambiguously observable; however, the stylus and setae of the left coxite appear to project from the terminal opening, which is indicative of the female identity of the specimen (along witht the triangular ventral segment VII).

**Male.** Unknown.

#### Diagnosis

**Generic placement.** *Tropirhinus palpebratus* [FZ2017] shares with (e.g.) *Tropirhinus elegans* (Guérin 1847) sec. Franz (2012) numerous phylogenetically informative traits inferred in Franz (2012) that substantiate this generic placement (see also Tables 1, 2, 3, 4, 5, 6). They include: 9(1): rostrum in lateral profile slightly arched and tumescent in mid region of dorsal surface; 16(1): rostrum dorsally mono- or tricarinate; 17(0): rostrum monocarinate, with one median, wide and rounded carina; 23(1): scrobe (of antenna) passing ventrad of eye in lateral profile; 31(0): head-rostrum transition in lateral profile continuous or only slightly angulate; 32(0): head in dorsal profile without a conspicuous postocular constriction; 34(0): head without an anteocular invagination; 58(2): metatibial apex with an outer bevel ("corbel enclosed"; see Thompson 1992); 67(1): posterior margins of elytra with an ante-apical, narrowly triangular projection; and 83(0): wings fully developed. The most parsimonious reconstructions furthermore postulates two wing properties that cannot be obversed in this amber inclusion, viz. 84(1): wings in proximal third with at least one patch of small, densely arranged denticles; and 85(2): patches of denticles in proximal region of wings distributed in rows along R (vein). This combination of character states is shared only between *Tropirhinus palpebratus* [FZ2017] and *Tropirhinus elegans* sec. Franz (2012) as coded in that latter analysis, and this correspondence of phylogenetically informative traits is the primary justification for assigning *Tropirhinus palpebratus* [FZ2017] to *Tropirhinus* [FZ2017].

We thereby assign to *Tropirhinus* [FZ2017] an expanded circumscription in comparison to (e.g.) *Tropirhinus* sec. O'Brien and Wibmer (1982), whose three members lack a postocular lobe and in turn have variously patterned metallic-colored maculae on the pronotum and elytra (see Guérin-Méneville 1847, Chevrolat 1877, Zhang et al. 2017). These and other apparent differences - e.g., only *Tropirhinus novemdecimpunctatus* (Fabricius 1781) sec. O'Brien and Wibmer (1982) has strongly protruding eyes - could be emphasized to justify the creation of a new genus-level name for the amber-preversed specimen under study. However, two kinds of considerations caution against this at present (see Vences et al. 2013). First, the characters and states that distinguish *Tropirhinus palpebratus* [FZ2017] from the other members of *Tropirhinus* [FZ2017] are frequently homoplasious in this greater lineage of Caribbean entimine weevils (as analyzed in Franz 2012 and Zhang et al. 2017). The presence or absence of a postocular lobe, or of postocular vibrissae, are variable traits within *Diaprepes* Schoenherr [FZ2017] (see also O'Brien and Kovarik 2001, Franz 2012). Metallic coloration patterns are often variable within and among the recognized members of (e.g.) *Exophthalmus* Schoenherr 1823 sec. Morrone (1999). Accordingly, our expanded genus-level concept *Tropirhinus* [FZ2017] entails primarily characters and states that appear to be phylogenetically 'labile' at low taxonomic levels. We consider this acceptable. Second, creating a new genus-level name for this specimen makes no tangible contribution to resolving the taxonomic identity of extant and closely related lineages, including members of *Compsoricus* Franz 2012 sec. Franz (2012), *Pachnaeus* Schoenherr 1826 sec. O'Brien and Wibmer (1982), *Tetrabothynus* Labram and Imhoff 1852 sec. O'Brien and Wibmer (1982), and the likely taxonomically misnamed *Exophthalmus quindecimpunctatus* (Olivier 1807) sec. Franz (2012) (therein incorrectly spelled) and *Exophthalmus roseipes* (Chevrolat 1876) sec. Franz (2012). The combination of an abundance of available genus-level names and still inadequate knowledge of the species-level diversity and phylogenetic relationships of the various aforementioned lineages (see also Zhang et al. 2017) makes it less appealing to create yet another genus-level name at this juncture.

Franz (2012) assigned *Tropirhinus* sec. Franz (2012) to the tribe Geonemini Gistel 1856 [non-focal], and this placement is not under taxonomic scrutiny here.

**Differential diagnosis.** *Tropirhinus palpebratus* [FZ2017], in addition to being extinct and recorded from Dominican amber, is readily distinguished from the extant members of *Tropirhinus* [FZ2017] by the presence of a small, postocular lobe (with a setal patch ventral thereof) and absence of metallic-colored pronotal and elytral maculae. Moreover, the eyes of *Tropirhinus palpebratus* [FZ2017] are more globular and protruded than those of *Tropirhinus elegans* sec. Franz (2012) and *Tropirhinus tredecimpunctutatus* (Guérin 1847) sec. O'Brien and Wibmer (1982), although those of *Tropirhinus novemdecimpunctatus* sec. O'Brien and Wibmer (1982) are similarly globular (see Chevrolat 1877; in particular plate IV, figure 4 therein). Lastly, *Tropirhinus palpebratus* [FZ2017] shows a smaller, only anteriorly extending pronotal sulcus, in contrast with a larger and more posteriorly extending pronotal impression that is flanked laterally by obtuse, rounded elevations, as present in other members of *Tropirhinus* [FZ2017]. Members of *Pachnaeus* sec. O'Brien and Wibmer (1982) have a wider rostrum and lack the posterior elytral projections, whereas those of *Tetrabothynus* sec. O'Brien and Wibmer (1982) have a postocular head constriction. Other close relatives (see Franz 2012) have distinctly different pronotal and elytral sculpture and coloration patterns; e.g. *Compsoricus* sec. Franz (2012) has large longitudinal carinae and *Exophthalmus quindecimpunctatus* sec. Franz (2012) has green metallic scales interspersed with distinct black maculae.

#### Etymology

The epithet - "eyelid, wink" (Brown 1956) - refers to the combination of the postocular lobe and the setal patch located ventral thereof - a set of traits that uniquely corresponds to *Tropirhinus palpebratus* [FZ2017] in relation to close relatives.

#### Distribution

*Tropirhinus palpebratus* [FZ2017] is known only from the examined Dominican amber inclusion ("USNM505319"; see Material) of the Burdigalian time period. The specific mine of origin for this inclusion is unknown.

#### Ecology

Unknown.

### Diaprepes anticus

Franz & Zhang [FZ2017] sp. n.

http://symbiota4.acis.ufl.edu/scan/portal/collections/individual/index.php?occid=25836761

urn:lsid:zoobank.org:act:92E4FDF2-B9E4-4441-AD74-261EDA89E661

#### Materials

- **Type status:** Holotype. **Occurrence:** catalogNumber: ARTSYS0000271; recordNumber: Woodruff #9774; recordedBy: R.E. Woodruff; individualCount: 1; sex: Female; lifeStage: Adult; preparations: Amber inclusion; disposition: USNM, on loan; otherCatalogNumbers: USNM505325; occurrenceID: 08bb94f8-fddc-4506-b454-34e7d27e5343; **Taxon:** scientificName: Diaprepes anticus; nameAccordingTo: Franz & Zhang 2017; namePublishedIn: Franz, N.M. & G. Zhang. 2017. Three new species of entimine weevils in Early Miocene amber from the Dominican Republic (Coleoptera: Curculionidae). Biodiversity Data Journal.; kingdom: Animalia; phylum: Arthropoda; class: Insecta; order: Coleoptera; family: Curculionidae; scientificNameAuthorship: Franz & Zhang, 2017; **Location:** country: Dominican Republic; stateProvince: La Vega; locality: Unknown mine; decimalLatitude: 19; decimalLongitude: -70.666667; geodeticDatum: WGS84; georeferencedBy: N.M. Franz; georeferenceSources: Google Earth; georeferenceVerificationStatus: Verfied by Curator; **Identification:** identifiedBy: N.M. Franz & G. Zhang; dateIdentified: 01/15/2017; identificationReferences: Franz, N.M. & G. Zhang. 2017. Three new species of entimine weevils in Early Miocene amber from the Dominican Republic (Coleoptera: Curculionidae). Biodiversity Data Journal.; **Event:** verbatimEventDate: Early Miocene, Burdigalian; habitat: Amber inclusion, Early Miocene (Burdigalian); **Record Level:** modified: 24/01/2017 18:31; rights: http://creativecommons.org/publicdomain/zero/1.0/; rightsHolder: United States National Museum; bibliographicCitation: Franz, N.M. & G. Zhang. 2017. Three new species of entimine weevils in Early Miocene amber from the Dominican Republic (Coleoptera: Curculionidae). Biodiversity Data Journal.; institutionID: USNM; collectionID: 0acac5fe-f0ec-4d9f-82f8-0dbb74888de2; institutionCode: SCAN; collectionCode: ARTSYS; ownerInstitutionCode: USNM; basisOfRecord: FossilSpecimen; source: http://symbiota4.acis.ufl.edu/scan/portal/collections/individual/index.php?occid=25836761

#### Description

**Figure 3.**
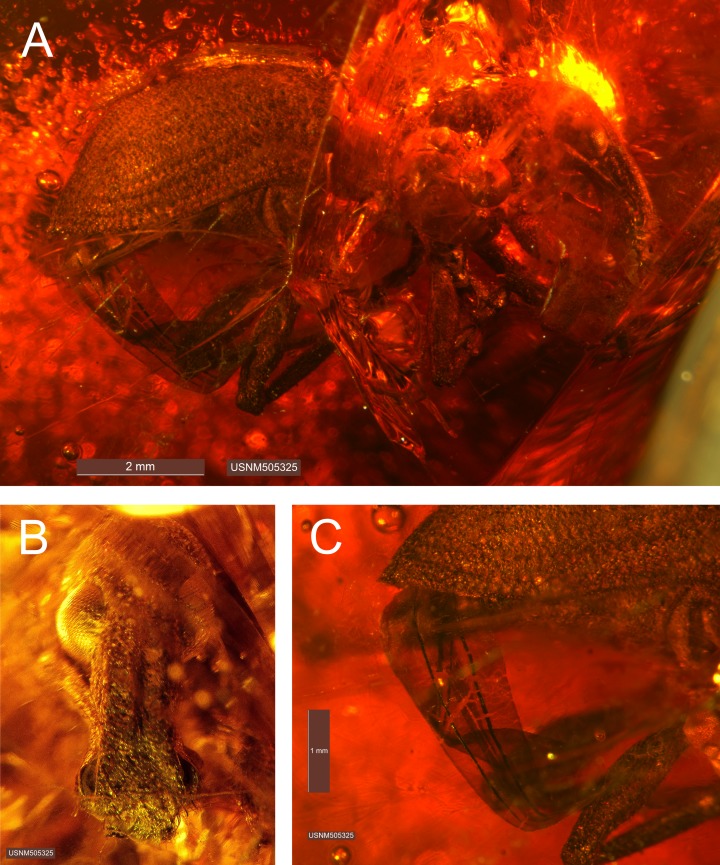
*Diaprepes anticus* Franz & Zhang 2017 **sp. n.** [FZ2017], female holotype, specimen USNM505325 (= ARTSYS000271). (A) Habitus, lateral view; (B) rostrum and head, frontal view; (C) lateral view, posterior aspect (thoracic appendages, abdomen).

**Female - habitus (Fig. 3).** Length 9.1 mm, width 3.8 mm; shape in dorsal view oval to elongate, length/width ratio 2.4, widest near mid region of elytra; shape in lateral view elongate to pyriform. Integument uniformly dark brown to black; surface punctate, though with larger, irregularly spaced and shaped concavities on pronotum; integument covered with setae and scales, most dense on elytra, less so on head (including rostrum) and legs; scales completely covering elytra, small, subcircular, overlapping, apparently predominatly pale in color though interspersed with green metallic scales, particularly along lateral regions of elytra and on the head and legs; setae short and linear, pale yellow, densely and regularly arranged, particularly on pronotum and elytra where setae are recurvate and directed mesally to posteriorly, setae longer, fine, aurate, and suberect on legs.

**Mouthparts.** Mandibles equilateral, asymmetrical, with 6-10 fine setae of variable length; mandibular scar positioned apicolaterally, projected. Maxillae with maxillary palps 3-segmented. Labium with prementum cordate, equilateral; labial palps apparently 3-segmented.

**Rostrum.** Length 1.45 mm, rostral/pronotal length ratio 0.75, rostral length/width ratio 1.8. Rostrum in dorsal view elongate, dorsolateral margins subparallel and weakly arcuate along posterior 2/3, expanded along anterior 1/3, anterodorsal margin weakly emarginate; epistoma with nasal plate (see Vaurie 1963) slightly angled in relation to posterior rostrum region, weakly depressed, V-shaped carina weakly projected, and posteriad thereof (at transition of nasal plate and remainder of rostrum) with a slight, transverse carina which is mesally interruped and posteriorly connected to a median, longitudinal carina (see Franz 2012: character 14[1]); dorsal surface tricarinate, with 1 stronger median and 2 weaker dorsolateral carinae, each carina narrowly rounded, glabrate, extending posteriorly to mid point between eyes, the paired dorsolateral carinae anteriorly slightly diverging (see Franz 2012: character 17[1]). Rostrum in lateral view sightly arcuate, width similar throughout; scrobe lateral, subrectate, passing over ventral region of eye and terminating near mid point of eye where the scrobe is continuous with the occipital suture (see Lyal 1995 and Franz 2012: character 28[1]); antennal insertion near anterior 1/4. Rostrum in ventral view with a long, triangular impression (see Franz 2012: character 29[2]). Antennae 11-segmented, covered with sparse metallic scales and fine, recurved setae; scape slender, slightly arcuate, extending to posterior margin of eye, and passing over eye (in idealized position); funicle 7-segmented, funicular antennomeres elongate, weakly clavate, increasingly shorter towards apex; club 3-segmented, elongate, similar in length to funicular antennomeres V-VII.

**Head.** Eyes large, globular (strongly roundly protruded), dorsolaterally positioned, separated by distance slightly shorter than anterior-to-posterior length of each eye; outline in lateral view elliptical (horizontally more extended), ventral margin less rounded.

**Thorax.** Pronotum in dorsal view equilateral, length/width ratio 1.5 (though challenging to observe due to the fossil's position in an inclusion with limited viewing access of the pronotum), pronotal/elytral length ratio 0.5; widest near posterior margin, lateral margins continously rounded and posteriorly diverging; surface punctate to foveate, with irregularly spaced and shaped concavities, ranging from subcircular to elongate to arcuate (see Franz 2012: character 45[1]), though no scales apparent therein; median sulcus absent. Pronotum in lateral view equilateral; anterolateral margins straight (without postocular lobe), presence of postocular vibrissae not unambiguously observable. Scutellum exposed by elytra, small, subcircular. Epipleura with mesepisternum triangular; mesepimeron dorsally oblique truncate; metepisternum narrowly linear, anteriorly widened; metepimeron entirely covered by elytron. Prothoracic ventrite with anterior transverse sulcus; procoxal cavities positioned near mid point, contiguous. Mesothoracic ventrite challenging to observe, though mesocoxal cavitities apparently narrowly separated. Metathoracic ventrite with median sulcus present as a large, transverse fovea positioned anteriad of posterior margin; metacoxal cavities separated by distance similar to width of each metacoxal cavity. Metendosternite not observed.

**Legs.** Prothoracic and metathoracic legs each slightly longer than mesothoracic legs (mesofemora shortest in comparison), highly similar to those of *Diaprepes abbreviatus* sec. Franz (2010a). Profemoral/pronotal length ratio 1.05; profemur ventrally inermous. Protibial/profemoral length ratio 1.4; protibia apically angulate-arcuate, width similar throughout, apex slightly expanded; anteromesal margin with 8-12 small, narrowly triangular teeth, each tooth distally with 1 brownish, spiniform seta; protibial apex weakly rounded, setal comb absent, promucro similar in length to protarsal claw; protarsus with tarsomere I slightly longer than II which is similar in length to III, yet I shorter than V; protarsal claws paired, separate, simple. Mesotibiae and metatibiae nearly straight, apically slightly expanded and weakly rounded; metatibial apex with an elliptical outer bevel ("corbel enclosed"; see Thompson 1992).

**Elytra.** Length/width ratio 1.5; widest near mid region; anterior margins jointly wider than posterior margin of pronotum (though challenging to observe along a crack in the amber inclusion), slightly sinuate; humeri present, rounded; lateral margins subparallel along anterior 1/2, therafter gradually and roundly converging, posterior edges narrow, actue, though not projected. Elytra in lateral view with dorsal outline weakly convex along anterior 3/4, thereafter (along posterior 1/4) with weakly angulate, straight declivity. Elytra with striae I-IX complete, stria X only apparent along anterior and posterior 1/3; striae slightly narrower than intervals; punctures separated by distance shorter than or similar to width of each puncture; intervals slightly and roundly elevated, no carinae apparent (as, e.g., in *Diaprepes abbreviatus* Franz 2010a); pale-colored and interspersed green metallic scales (the latter particularly in lateral regions) covering elytra densely and homogenously, with no maculae apperent; with short, linear to spatulae, pale yellow to light brown, regularly spaced, mesally to posteriorly directed setae throughout elytral surface.

**Wings.** Present, and visible in part (apical 1/2) since the specimen had its wings extended prior to its preservation in amber; veins RP1 and RP2 apparent (see Oberprieler et al. 2014), and interjacently with a large, longitudinal, brown macula.

**Abdomen.** Venter with segments III and IV jointed (see Thompson 1992 for segment homology), III slightly longer than IV, and separated by sinuate suture; V-VII separate; V and VI jointly as long as IV, posterior margins elevated-projected; VII slightly longer than III, triangular, posteriorly narrowly rounded, subacute (see Franz 2012: character 86[1]); all segments densely covered with whitish, appressed scales (absent laterally in III-V), VII posteriorly with long, suberect, aurate setae. Pygidium posteriorly narrowly converging, subacute, covered by elytra.

**Terminalia.** Not externally visible; however, the triangular, posteriorly narrowly projected ventral segment VII is indicative of this specimen being female (see Franz 2010a, Franz 2012).

**Male.** Unknown.

#### Diagnosis

**Generic placement.** *Diaprepes anticus* [FZ2017] shares with (e.g.) *Diaprepes maugei* (Boheman 1840) sec. Franz 2012 numerous phylogenetically informative traits inferred in Franz 2012 that substantiate this generic placement (see also Tables 1, 2, 3, 4, 5, 6). They include: 14(1): rostrum with epistoma (see Vaurie 1963) posteriorly separated from remainder of rostrum by a slight transverse carina, which is mesally interrupted and posteriorly connected to a median longitudinal carina; 16(1): rostrum dorsally mono- or tricarinate; 17(1) rostrum tricarinate, with a characteristic combination of one median carina and two (dorso-) lateral, apically slightly diverging carinae, each carina narrow, moderately sharp; 23(0): scrobe (of antenna) passing over eye in lateral profile; 28(1): rostrum on ventral side with occipital sutures (see Lyal 1995) posteriorly moderately wide and deep, oriented horizontally, and extending to ventral midpoint of eye; 29(2): rostrum on ventral side with long, narrowly triangular impression flanked by hypostomal-labial sutures (see Lyal 1995); 45(1): pronotum in dorsal profile with small, shallow, densely arranged, irregularly shaped and spaced concavities, covered with varying numbers of small, elongate, appressed scales; 58(2): metatibial apex with an outer bevel ("corbel enclosed"; see Thompson 1992); 64(0): elytra in dorsal provide with strial intervals not roundly elevated; 83(0): wings fully developed; and 86(1): female with sternum VII of venter in ventral profile posteriorly sharply and narrowly triangular, lateral margins straight. This combination of character states is shared only between *Diaprepes anticus* [FZ2017], *Diaprepes famelicus* (Olivier 1790) sec. Franz 2012, *Diaprepes marginicollis* Chevrolat 1880 sec. Franz 2012, and *Diaprepes maugei* sec. Franz 2012 as coded in that latter analysis, and therfore justifies the placement of *Diaprepes anticus* [FZ2017] within *Diaprepes* [FZ2017].

Franz 2012 assigned *Diaprepes* sec. Franz 2012 to the tribe Eustylini Lacordaire 1863 [non-focal], and this placement is not under taxonomic scrutiny here.

**Differential diagnosis.** *Diaprepes anticus* [FZ2017], in addition to being extinct and recorded from Dominican amber, is readily distinguished from extant members of *Diaprepes* [FZ2017] by the absence of postocular vibrissae (character 48[0]), the absence of variously extended, rounded, and glabrate elytra carinae (character 64[0]), and the absence of striped elytral regions with intermixed appressed and suberect scales (character 79[0]; all characters and states according to Franz 2012). *Diaprepes anticus* [FZ2017] most closely resembles *Diaprepes famelicus* sec. Franz 2012 and in particular *Diaprepes maugei* sec. Franz 2012 with which is shares metallic scale coloration (see also O'Brien and Kovarik 2001). Nevertheless, the pronotal and elytral scale and setal patterns of *Diaprepes anticus* [FZ2017] are diagnostic by virtue of combining densely and homogenously arranged pale scales with interspersed metallic scales and abundant, short, spatulate setae. The apparent transverse rostral carina more roundly protruded eyes, and less posteriorly acute female ventral segment VII further distinguish *Diaprepes anticus* [FZ2017] from the aforementioned and presumed close relatives.

#### Etymology

The epithet - "in front, foremost" (Brown 1956) - refers to *Diaprepes anticus* [FZ2017] being oldest documented member of *Diaprepes* [FZ2017] .

#### Distribution

*Diaprepes anticus* [FZ2017] is known only from the examined Dominican amber inclusion ("USNM505325"; see Material) of the Burdigalian time period. The specific mine of origin for this inclusion is unknown.

#### Ecology

Unknown.

## Discussion

### Taxononomic concept alignments

We present consistent Region Connection Calculus (RCC-5) alignments and visualizations of current and preceding taxonomic concepts that are relevant to our newly recognized names and entities. The process of generating such alignments is described in detail in Franz and Cardona-Duque (2013), Franz et al. (2015), Jansen and Franz (2015), Franz et al. (2016a), Franz et al. (2016b), and we refer to these publications for additional explanation. In each case, we utilized the current version (August, 2016) of the open source Euler/X toolkit (Chen et al. 2014, available at https://github.com/EulerProject/). One novel aspect of the alignments and visualizations is that 3-5 taxonomic concept hierarchies are processed simultaneously, whereas previous analyses were limited to pairwise alignments.

**Figure 4.**
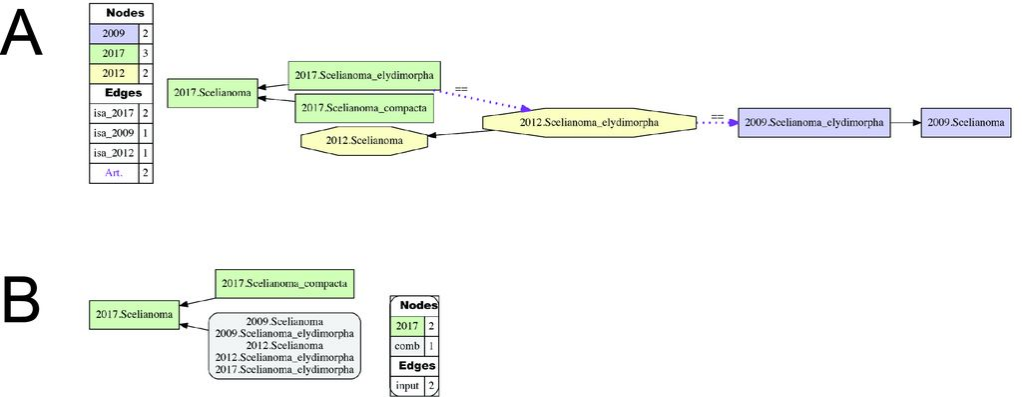
(A) Input visualization and (B) alignment visualization for the taxonomic concept alignment of Scelianoma Franz and Girón 2009 sec. auctorum. See also Suppl. materials 2, 3, 4, 5. In this and in succeeding visualizations, we use the convention "2012.Scelianoma" (etc.) to abbreviate the taxonomic concept label *Scelianoma* Franz and Girón 2009 sec. Franz (2012). In the input visualization (A), given parent-child relationships within a single taxonomy are symbolized with solid black arrows, whereas provided RCC-5 articulations are shown with dashed magenta arrows. The symbols used for expert-provided RCC-5 articulations are: == for congruence, > for proper inclusion, < for inverse proper inclusion, >< for overlap, and ! for exclusion. Taxonomic concepts are color-coded in the input visualizations to reflect their unique sources of authorship; whereas in the alignment visualizations they retain these colors only if they are (also) taxonomically unique, i.e., not congruent with other taxonomic concepts. Multiple congruence taxonomic concepts are represented in grey rectangles with rounded corners. In this particular alignment visualization (B), only solid black arrows appear, indicating proper inclusion that was inferred through an early-stage reasoning process. In other alignment visualizations, red solid arrows indicate proper inclusion that was inferred through a late-state reasoning process, and dashed blue lines significant overlap.

**Alignment of Scelianoma Franz and Girón 2009 sec. auctorum.** (Fig. 4; Suppl. materials 2, 3, 4, 5Suppl. materials 2, 3, 4, 5). This alignment is straightforward, and includes the classifications of Franz and Girón (2009), Franz (2012), and the present study (2016). Accordingly, the recognition of *Scelianoma compacta* [FZ2017] contributes to a more inclusive circumscription of *Scelianoma* [FZ2017] in comparison with preceding treatments. The differences between the two members of *Scelianoma* [FZ2017] are reviewed in the corresponding "differential diagnosis" section.

**Figure 5.**
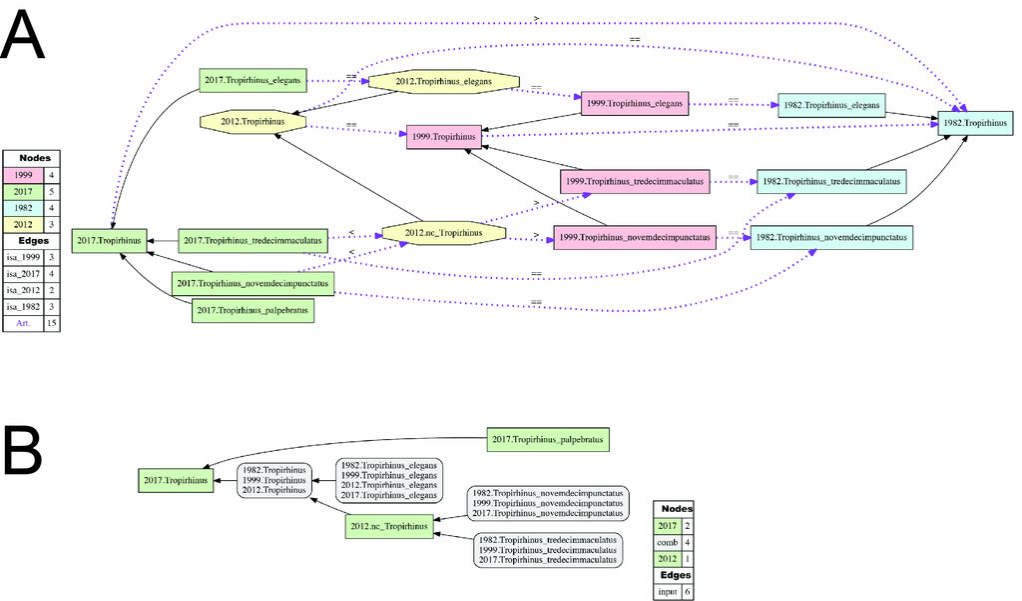
(A) Input visualization and (B) alignment visualization for the taxonomic concept alignment of Tropirhinus Schoenherr 1823 sec. auctorum. See also Suppl. materials 6, 7, 8, 9.

**Alignment of Tropirhinus Schoenherr 1823 sec. auctorum.** (Fig. 5; Suppl. materials 6, 7, 8, 9). We provide an alignment of the following four classifications: O'Brien and Wibmer (1982), Morrone (1999), Franz (2012), and the present study (2016). As in the preceding alignment, recognizing *Tropirhinus palpebratus* [FZ2017] yields a more inclusive concept *Tropirhinus* [FZ2017] in relation to the preceding treatments of genus-level concepts using this name. The salient features distinguishing *Tropirhinus palpebratus* [FZ2017] from other members of *Tropirhinus* [FZ2017] are reviewed in the corresponding "generic placement" section. The immediately preceding analysis (2012) only examined *Tropirhinus elegans* sec. Franz (2012), and our alignment accounts for this incomplete sampling by relaxing the coverage constraint ("2012.nc_Tropirhinus") for *Tropirhinus* sec. Franz (2012). This means that the region represented by this parent concept need not be exclusively defined by its single child. We can thereby express "intensional congruence" with preceding genus-level concepts (see Franz et al. 2015).

**Figure 6.**
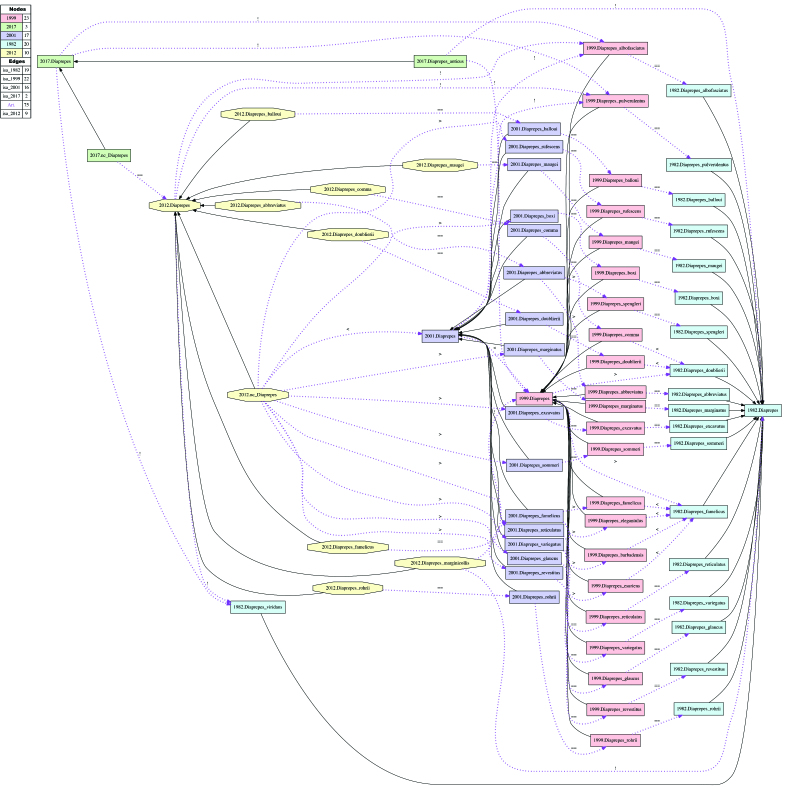
Input visualization for the taxonomic concept alignment of Diaprepes Schoenherr 1823 sec. auctorum. See also Suppl. materials 10, 11, 12, 13.

**Figure 7.**
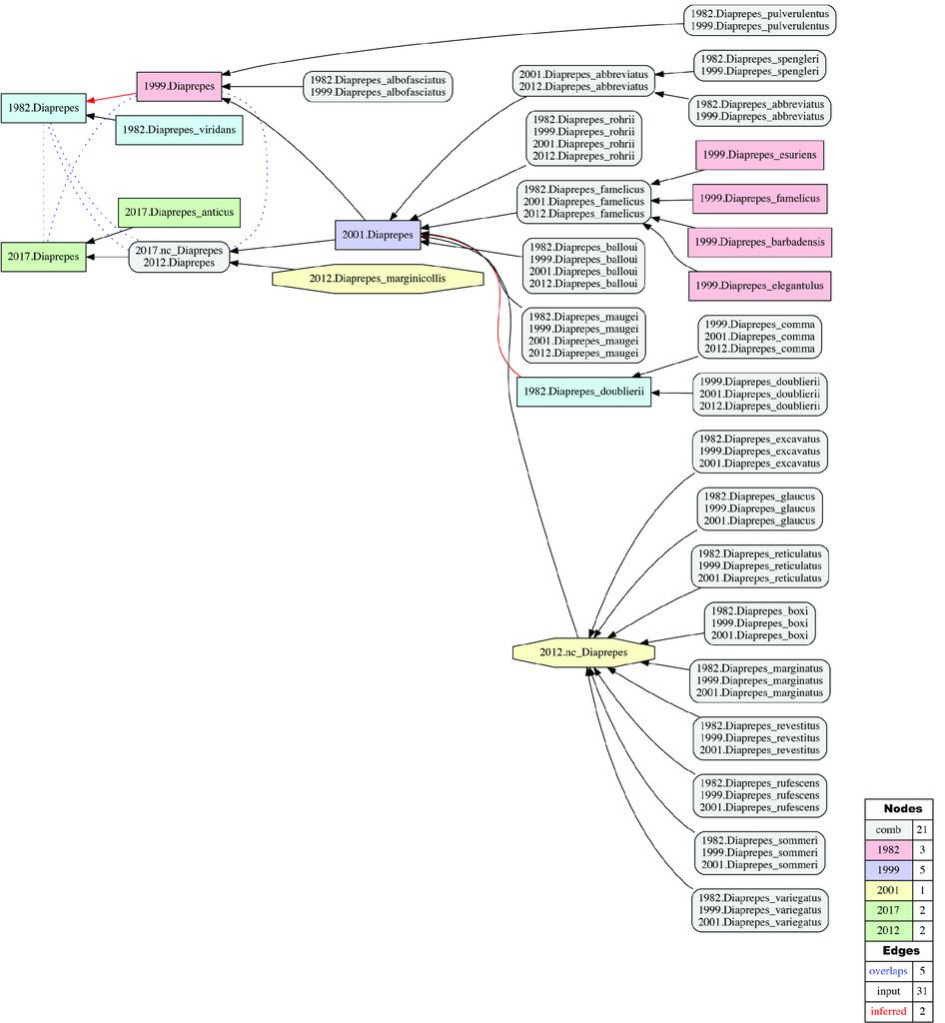
Alignment visualization for the taxonomic concept alignment of Diaprepes Schoenherr 1823 sec. auctorum. See also Suppl. materials 10, 11, 12, 13.

**Alignment of Diaprepes Schoenherr 1823 sec. auctorum.** (Figs 6, 7; Suppl. materials 10, 11, 12, 13). This alignment is the most complex due to the number of taxonomic concepts represented - 73 in total - and frequent changes of recognized species-level entities across treatments as a result of (1) inclusion in or (2) exclusion from Diaprepes (sec. auctorum), or (3) synonymization of species-level entities within Diaprepes (sec. auctorum), modeled as proper inclusion in our RRC-5 alignment (e.g., 2001.Diaprepes_famelicus > 1999.Diaprepes_famelicus; see also Franz et al. 2016b). The following five classifications are aligned: O'Brien and Wibmer (1982), Morrone (1999), O'Brien and Kovarik (2001), Franz (2012), and the present study (2016). At the nominal genus level, the alignment yields five taxonomically incongruent regions labeled with *Diaprepes*. We relax the coverage constraint for *Diaprepes* sec. Franz (2012) and *Diaprepes* [FZ2017], given that each genus-level concept is 'purposefully' undersampled at the child level. Under these constraints, *Diaprepes* [FZ2017] properly includes (>) two immediately predecing concepts *Diaprepes* sec. Franz (2012) and *Diaprepes* sec. O'Brien and Kovarik (2001), and overlaps with (><) earlier concepts *Diaprepes* sec. Morrone (1999) and *Diaprepes* sec. O'Brien and Wibmer (1982) which entailed entities subsequently assigned to *Exophthalmus* sec. Franz (2012).

### Historical biogeographic implications

The recognition of the three Miocene-based fossils *Scelianoma compacta* [FZ2017], *Tropirhinus palpebratus* [FZ2017], and *Diaprepes anticus* [FZ2017] suggests that the corresponding weevil lineages are longstanding members of a diversified and specialized West Indian weevil fauna (see also Poinar 2010, Poinar and Legalov 2015, Poinar et al. 2013). The description of *Scelianoma compacta* [FZ2017] in particular indicates that the historical range of *Scelianoma* [FZ2017] extends beyond southwestern Puerto Rico (Franz and Girón 2009) to include Hispaniola. Thus we may posit that *Scelianoma* [FZ2017] was historically more widespread in the Greater Antilles than reflected in the present. Most extant members of *Diaprepes* [FZ2017], in turn, occur throughout the Lesser Antilles, and the presence of *Diaprepes anticus* [FZ2017] in Hispaniola during the Early Miocene may be indicative of an eastward directed process of island colonization and likely speciation. In combination with time-calibrated phylogenetic trees, the herein presented data will facilitate more reliable, parametric reconstructions of historical biographic processes thought to have played a prominent role in the diversification of the West Indian and Neotropical mainland broad-nosed weevil lineages (Zhang et al. 2017).

### Biodiversity informatics

This study has benefitted from a recent, relevant, and arguably thorough cladistic analysis (Franz 2012) , which in turn has provided an inferential framework for placing and naming the newly perceived taxa at into specific genus-level groups. In doing so, we have also promoted a practice of speaking precisely - in terms of making the contextuality of each taxonomic name usage explicit via taxonomic concept labels, assigning speaker roles by either authoring new or citing published taxonomic concepts, and by providing RCC-5 articulations to express the apparent congruence or non-congruence between the newly and previously recognized taxonomic entities. An obvious cororally of this approach is that we acknowledge the validity of our taxonomic inferences to be potentially epheremal. Hence we wish to explore syntatic and semantic solutions to the challenge of building open-ended biodiversity knowledge systems that can represent expert taxonomic knowledge published at a given time, as well as integrate evolving taxonomic knowledge over time, where much of the integration work is facilitated at scale by logic-based services (Franz et al. 2016b, Franz et al. 2016c, Franz et al. 2016a).

## Supplementary Material

Supplementary material 1 Expanded cladistic character matrix of Franz (2012), including Scelianoma compacta [FZ2017], Tropirhinus palpebratus [FZ2017], and Diaprepes anticus [FZ2017] Data type: Saved as text file (.txt), though originally in NONA file format (.ss)
File: oo_119552.csv Franz, N.M., Zhang, G.

Supplementary material 2 Euler/X input data file for the taxonomic concept alignment of ﻿Scelianoma﻿ Franz and Girón 2009 sec. auctorum Data type: Euler/X input data file (.txt)
Brief description: Euler/X input data file for the alignment of *Scelianoma* sec. 2009, 2012, and 2017.
File: oo_119551.txt Franz, N.M., Zhang, G.

Supplementary material 3 Euler/X output - set of Maximally Informative Relations (MIR) - taxonomic concept alignment of Scelianoma Franz and Girón 2009 sec. auctorum Data type: Comma separated values file (.csv) with RCC-5 ﻿taxonomic concept articulations
Brief description: Euler/X output of 16 Maximally Informative Relations inferred for the *Scelianoma* alignment
File: oo_119553.csv Franz, N.M., Zhang, G.

Supplementary material 4 Euler/X input visualization - taxonomic concept alignment of ﻿Scelianoma﻿ Franz and Girón 2009 sec. auctorum Data type: PDF of Euler/X input visualization for the ﻿Scelianoma﻿ alignment
File: oo_119554.pdf Franz, N.M., Zhang, G.

Supplementary material 5 Euler/X alignment visualization - taxonomic concept alignment of ﻿Scelianoma﻿ Franz and Girón 2009 sec. auctorum Data type: PDF of Euler/X alignment visualization for the ﻿Scelianoma﻿ alignment
File: oo_119555.pdf Franz, N.M., Zhang, G.

Supplementary material 6 Euler/X input data file for the taxonomic concept alignment o﻿f Tropirhinus Schoenherr 1823 sec. auctorum Data type: Euler/X data input text file (.txt)
Brief description: Euler/X input data file for the alignment of *Tropirhinus* sec. 1982, 1999, 2012, and 2017.
File: oo_119556.txt Franz, N.M., Zhang, G.

Supplementary material 7 Euler/X output - set of Maximally Informative Relations (MIR) - taxonomic concept alignment of ﻿Tropirh﻿inus﻿ Schoenherr 1823 sec. auctorum Data type: Comma separated values file (.csv) with RCC-5 taxonomic concept articulations
Brief description: Euler/X output of 95 Maximally Informative Relations inferred for the *Tropirhinus* alignment
File: oo_119558.csv Franz, N.M., Zhang, G.

Supplementary material 8 Euler/X input visualization - taxonomic concept alignment of Tropirhinus Schoenherr 1823 sec. auctorum Data type: PDF of Euler/X input visualization for the ﻿Tropirhinus﻿ alignment﻿
File: oo_119559.pdf Franz, N.M., Zhang, G.

Supplementary material 9 Euler/X alignment visualization - taxonomic concept alignment of Tr﻿opirhinus Schoenherr 1823 sec. auctorum Data type: PDF of Euler/X alignment visualization for the ﻿Tropirhinus﻿ alignment﻿
File: oo_119560.pdf Franz, N.M., Zhang, G.

Supplementary material 10 Euler/X input data file for the taxonomic concept alignment of ﻿Diaprepes﻿ Schoenherr 1823 sec. auctorum Data type: Euler/X input data text file (.txt.)
Brief description: Euler/X input data file for the alignment of *Diaprepes* sec. 1982, 1999, 2001, 2012, and 2017.
File: oo_119561.txt Franz, N.M., Zhang, G.

Supplementary material 11 Euler/X out - set of Maximally Informative Relation (MIR) - taxonomic concept alignment of Diaprepes Schoenherr 1823 sec. auctorum Data type: Comma separated values file (.csv) with RCC-5 taxonomic concept articulations
Brief description: Euler/X output of 2001 Maximally Informative Relations inferred for the *Diaprepes* alignment
File: oo_119562.csv Franz, N.M., Zhang, G.

Supplementary material 12 Euler/X input visualization - taxonomic concept alignment of ﻿Diaprepes ﻿Schoenherr 1823 sec. auctorum Data type: PDF of Euler/X input visualization for the ﻿Diaprepes﻿ alignment
File: oo_119563.pdf Franz, N.M., Zhang, G.

Supplementary material 13 Euler/X alignment visualization - taxonomic concept alignment of Diaprepes Schoenherr 1823 sec. auctorum Data type: PDF of Euler/X alignment visualization for the Diaprepes alignment
File: oo_119564.pdf Franz, N.M., Zhang, G.

XML Treatment for Scelianoma compacta

XML Treatment for Tropirhinus palpebratus

XML Treatment for Diaprepes anticus

## Figures and Tables

**Table 1.** Extension of the character matrix published in Appendix 1 of Franz (2012), with character/state codings 1-25 for *Scelianoma compacta* [FZ2017], *Tropirhinus palpebratus* [FZ2017], and *Diaprepes anticus* [FZ2017]. "-" means "inapplicable", and "?" means "missing information". See Franz (2012) for additional explanation.

**Label / character**	1	2	3	4	5	6	7	8	9	10	11	12	13	14	15	16	17	18	19	20	21	22	23	24	25
***Scelianoma compacta* [FZ2017]**	0	0	2	0	0	0	1	0	0	0	0	0	0	0	0	0	–	1	0	0	0	0	0	0	0
***Tropirhinus palpebratus* [FZ2017]**	0	0	2	0	0	0	1	0	1	0	0	0	0	0	0	1	0	0	0	0	0	0	1	0	0
***Diaprepes anticus* [FZ2017]**	0	0	2	0	0	0	1	0	0	0	0	0	0	1	0	1	1	0	0	0	0	0	0	0	0

**Table 2.** Extension of the character matrix published in Appendix 1 of Franz (2012), with character/state codings 26-50 for *Scelianoma compacta* [FZ2017], *Tropirhinus palpebratus* [FZ2017], and *Diaprepes anticus* [FZ2017]. "-" means "inapplicable", and "?" means "missing information". See Franz (2012) for additional explanation.

**Label / character**	26	27	28	29	30	31	32	33	34	35	36	37	38	39	40	41	42	43	44	45	46	47	48	49	50
***Scelianoma compacta* [FZ2017]**	1	0	0	0	0	0	0	0	1	1	0	0	0	0	0	0	0	0	0	0	–	–	0	?	?
***Tropirhinus palpebratus* [FZ2017]**	0	0	0	0	0	0	0	0	0	0	0	0	0	0	0	0	0	0	0	0	–	–	0	?	?
***Diaprepes anticus* [FZ2017]**	0	0	1	2	0	0	0	0	0	0	0	0	0	0	0	0	0	0	0	1	–	–	0	?	?

**Table 3.** Extension of the character matrix published in Appendix 1 of Franz (2012), with character/state codings 51-75 for *Scelianoma compacta* [FZ2017], *Tropirhinus palpebratus* [FZ2017], and *Diaprepes anticus* [FZ2017]. "?" means "missing information". See Franz (2012) for additional explanation.

**Label / character**	51	52	53	54	55	56	57	58	59	60	61	62	63	64	65	66	67	68	69	70	71	72	73	74	75
***Scelianoma compacta* [FZ2017]**	0	0	0	0	0	0	0	2	0	0	0	1	0	0	0	1	0	0	0	0	–	–	0	–	0
***Tropirhinus palpebratus* [FZ2017]**	0	0	0	0	0	0	0	2	0	0	0	0	0	0	0	0	1	0	0	0	–	–	0	–	0
***Diaprepes anticus* [FZ2017]**	0	0	0	0	0	0	0	2	0	0	0	0	0	0	0	0	0	0	0	0	–	–	0	–	0

**Table 4.** Extension of the character matrix published in Appendix 1 of Franz (2012), with character/state codings 76-100 for *Scelianoma compacta* [FZ2017], *Tropirhinus palpebratus* [FZ2017], and *Diaprepes anticus* [FZ2017]. "?" means "missing information". See Franz (2012) for additional explanation.

**Label / character**	76	77	78	79	80	81	82	83	84	85	86	87	88	89	90	91	92	93	94	95	96	97	98	99	100
***Scelianoma compacta* [FZ2017]**	0	0	0	0	0	–	–	1	–	–	0	0	0	?	?	?	?	?	?	?	?	?	?	?	?
***Tropirhinus palpebratus* [FZ2017]**	0	0	0	0	0	0	0	?	?	?	0	0	0	?	?	?	?	?	?	?	?	?	?	?	?
***Diaprepes anticus*** [**FZ2017**]	0	0	0	0	0	–	–	0	0	0	1	0	0	?	?	?	?	?	?	?	?	?	?	?	?

**Table 5.** Extension of the character matrix published in Appendix 1 of Franz (2012), with character/state codings 101-125 for *Scelianoma compacta* [FZ2017], *Tropirhinus palpebratus* [FZ2017], and *Diaprepes anticus* [FZ2017]. "?" means "missing information". See Franz (2012) for additional explanation.

**Label / character**	101	102	103	104	105	106	107	108	109	110	111	112	113	114	115	116	117	118	119	120	121	122	123	124	125
***Scelianoma compacta* [FZ2017]**	?	?	?	?	?	?	?	?	?	?	?	?	?	?	?	?	?	?	?	?	?	?	?	?	?
***Tropirhinus palpebratus* [FZ2017]**	?	?	?	?	?	?	?	?	?	?	?	?	?	?	?	?	?	?	?	?	?	?	?	?	?
***Diaprepes anticus* [FZ2017]**	?	?	?	?	?	?	?	?	?	?	?	?	?	?	?	?	?	?	?	?	?	?	?	?	?

**Table 6.** Extension of the character matrix published in Appendix 1 of Franz (2012), with character/state codings 126-143 for *Scelianoma compacta* [FZ2017], *Tropirhinus palpebratus* [FZ2017], and *Diaprepes anticus* [FZ2017]. "?" means "missing information". See Franz (2012) for additional explanation.

**Label / character**	126	127	128	129	130	131	132	133	134	135	136	137	138	139	140	141	142	143
***Scelianoma compacta* [FZ2017]**	?	?	?	?	?	?	?	?	?	?	?	?	?	?	?	?	?	?
***Tropirhinus palpebratus* [FZ2017]**	?	?	?	?	?	?	?	?	?	?	?	?	?	?	?	?	?	?
***Diaprepes anticus* [FZ2017]**	?	?	?	?	?	?	?	?	?	?	?	?	?	?	?	?	?	?
