# Supplementary figures and images for: Three new species of entimine weevils in Early Miocene amber from the Dominican Republic (Coleoptera: Curculionidae)
Source: Biodivers Data J. 2017 Feb 3;(5):e10469. doi: 10.3897/BDJ.5.e10469 (PMC5345054; doi:10.3897/BDJ.5.e10469)

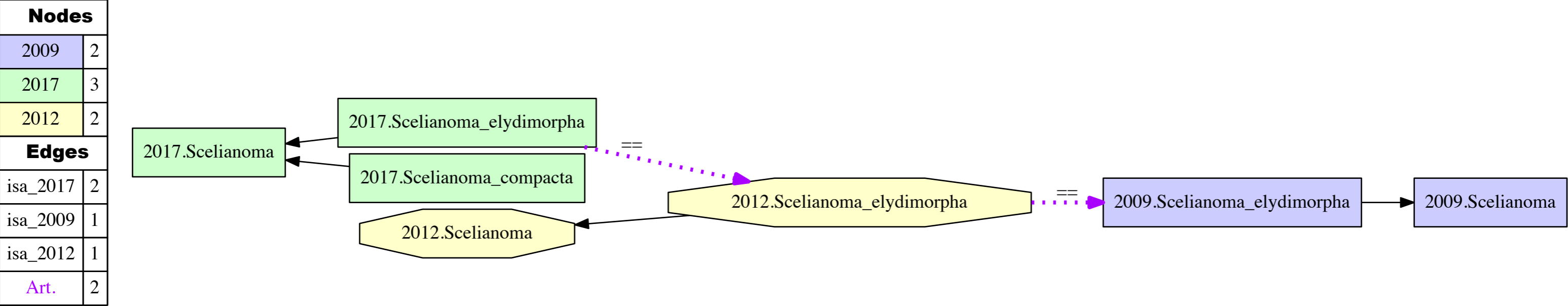

Supplement: Supplementary material 4 — Euler/X input visualization - taxonomic concept alignment of ﻿Scelianoma﻿ Franz and Girón 2009 sec. auctorum [file bdj-05-e10469-s004.pdf]

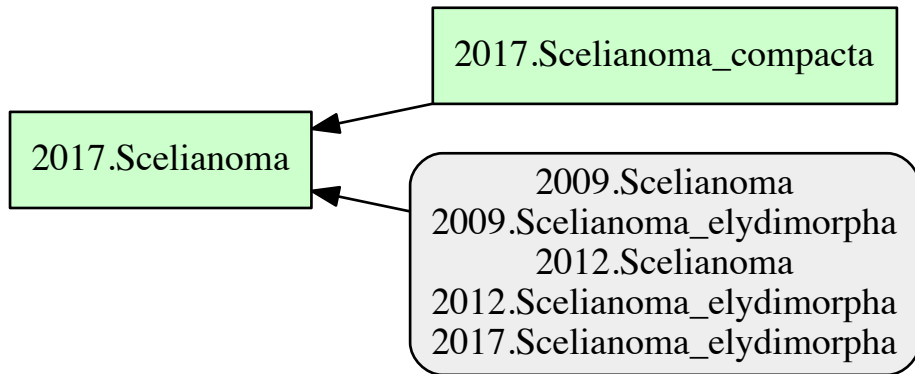

| Nodes |   |
|-------|---|
| 2017  | 2 |
| comb  | 1 |
| Edges |   |
| input | 2 |

Supplement: Supplementary material 5 — Euler/X alignment visualization - taxonomic concept alignment of ﻿Scelianoma﻿ Franz and Girón 2009 sec. auctorum [file bdj-05-e10469-s005.pdf]

| Nodes    |    |
|----------|----|
| 1999     | 4  |
| 2017     | 5  |
| 1982     | 4  |
| 2012     | 3  |
| Edges    |    |
| isa_1999 | 3  |
| isa_2017 | 4  |
| isa_2012 | 2  |
| isa_1982 | 3  |
| Art.     | 15 |

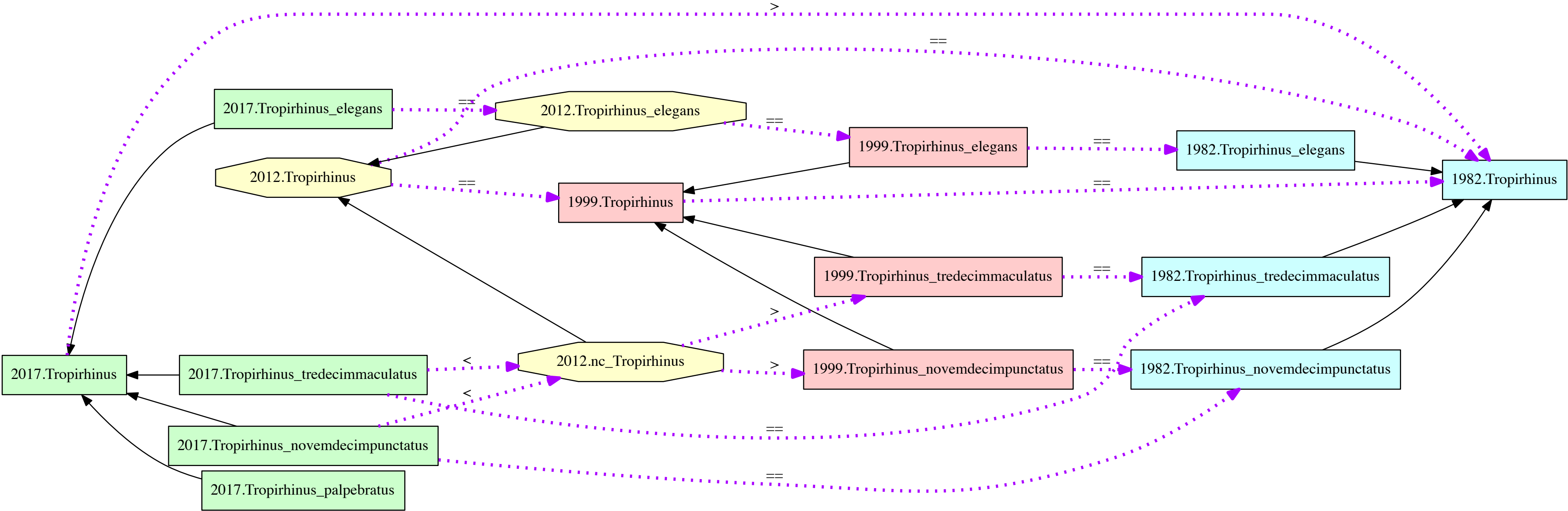

Supplement: Supplementary material 8 — Euler/X input visualization - taxonomic concept alignment of Tropirhinus Schoenherr 1823 sec. auctorum [file bdj-05-e10469-s008.pdf]

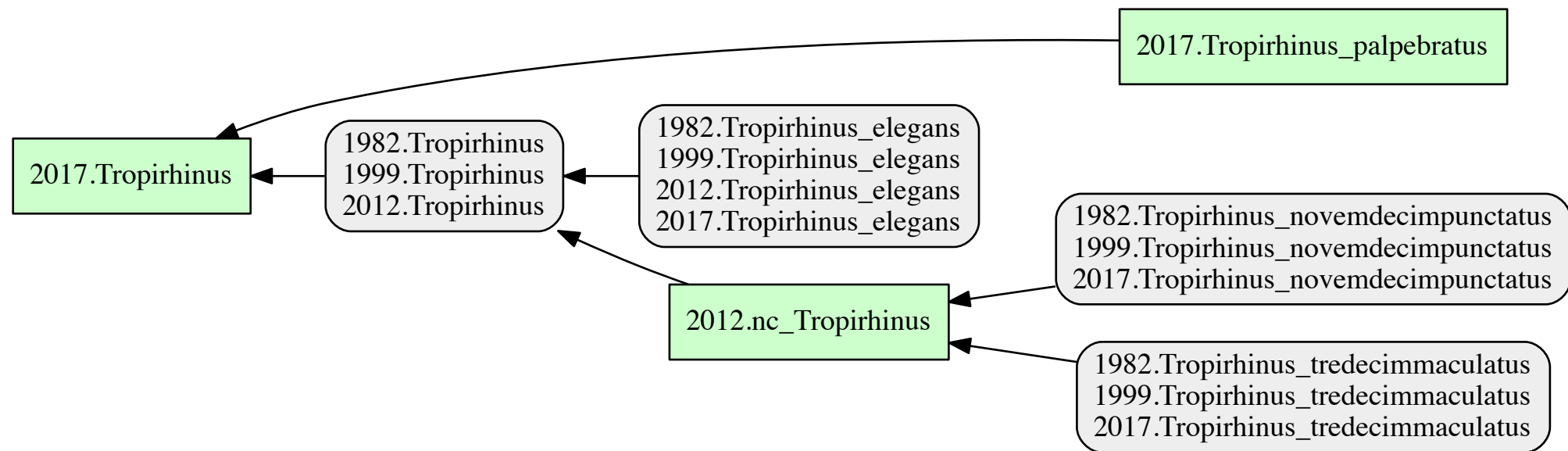

| Nodes |   |
|-------|---|
| 2017  | 2 |
| comb  | 4 |
| 2012  | 1 |
| Edges |   |
| input | 6 |

Supplement: Supplementary material 9 — Euler/X alignment visualization - taxonomic concept alignment of Tr﻿opirhinus Schoenherr 1823 sec. auctorum [file bdj-05-e10469-s009.pdf]

| Nodes    |    |
|----------|----|
| 1999     | 23 |
| 2017     | 3  |
| 2001     | 17 |
| 1982     | 20 |
| 2012     | 10 |
| Edges    |    |
| isa_1982 | 19 |
| isa_1999 | 22 |
| isa_2001 | 16 |
| isa_2017 | 2  |
| Art.     | 75 |
| isa_2012 | 9  |

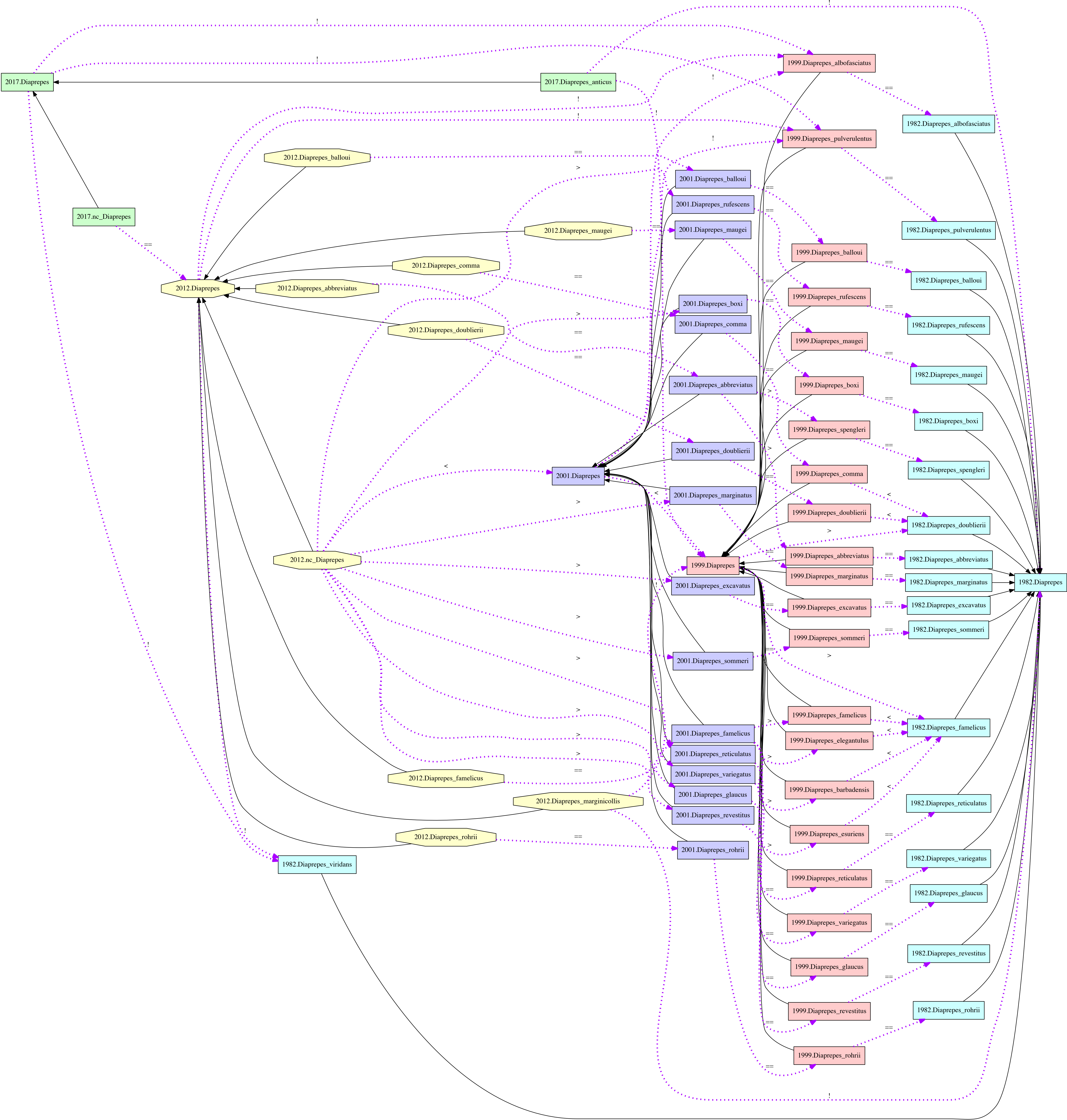

Supplement: Supplementary material 12 — Euler/X input visualization - taxonomic concept alignment of ﻿Diaprepes ﻿Schoenherr 1823 sec. auctorum [file bdj-05-e10469-s012.pdf]
