# Supplementary material for: Three new species of entimine weevils in Early Miocene amber from the Dominican Republic (Coleoptera: Curculionidae)
Source: Biodivers Data J. 2017 Feb 3;(5):e10469. doi: 10.3897/BDJ.5.e10469 (PMC5345054; doi:10.3897/BDJ.5.e10469)
Supplement: Supplementary material 13 — Euler/X alignment visualization - taxonomic concept alignment of Diaprepes Schoenherr 1823 sec. auctorum [file bdj-05-e10469-s013.pdf]

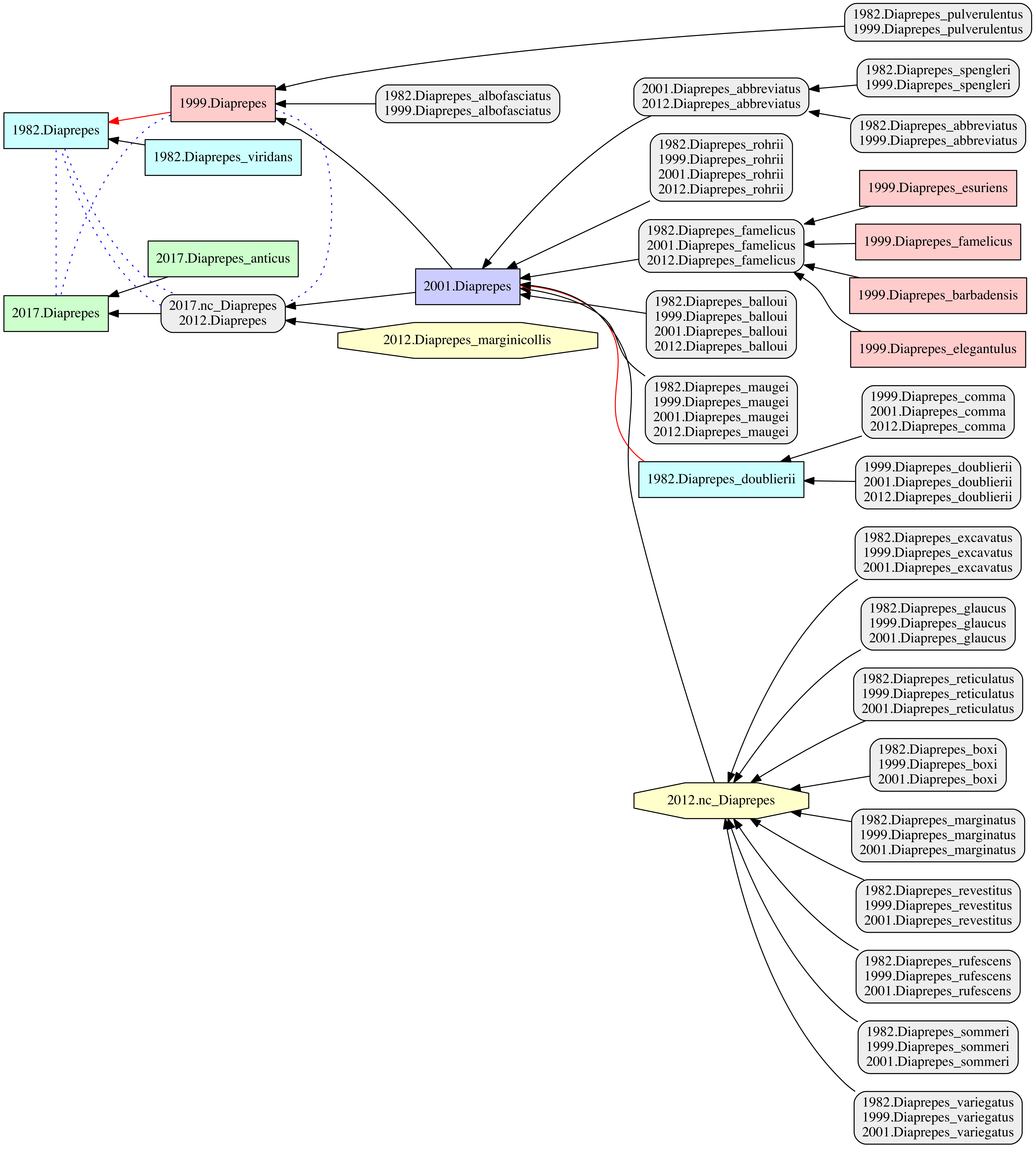

**Nodes**	
comb	21
1982	3
1999	5
2001	1
2017	2
2012	2
**Edges**	
overlaps	5
input	31
inferred	2
